# Clinical assessment of patients with chest pain; a systematic review of predictive tools

**DOI:** 10.1186/s12872-016-0196-4

**Published:** 2016-01-20

**Authors:** Luis Ayerbe, Esteban González, Valentina Gallo, Claire L. Coleman, Andrew Wragg, John Robson

**Affiliations:** Centre for Primary Care and Public Health, Queen Mary University of London, Yvonne Carter Building, 58 Turner Street, London, E1 2AB UK; Family Medicine Unit, Department of Medicine, Autónoma University of Madrid, Madrid, Spain; Department of Cardiology, Barts Health NHS Trust, London, UK

**Keywords:** Chest pain, Myocardial ischaemia, Risk assessment, Primary health care, Cardiology, Emergency medicine

## Abstract

**Background:**

The clinical assessment of patients with chest pain of recent onset remains difficult. This study presents a critical review of clinical predictive tools for the assessment of patients with chest pain.

**Methods:**

Systematic review of observational studies and estimation of probabilities of coronary artery disease (CAD) in patients with chest pain. Searches were conducted in PubMed, Embase, Scopus, and Web of Science to identify studies reporting tools, with at least three variables from clinical history, physical examination or ECG, produced with multivariate analysis, to estimate probabilities of CAD in patients with chest pain of recent onset, published from inception of the database to the 31^st^ July 2015. The references of previous relevant reviews were hand searched. The methodological quality was assessed with standard criteria. Since the incidence of CAD has changed in the past few decades, the date of publication was acknowledged to be relevant in order to use the tool in clinical practice, and more recent papers were considered more relevant. Probabilities of CAD according to the studies of highest quality were estimated and the evidence provided was graded.

**Results:**

Twelve papers were included out of the 19126 references initially identified. The methodological quality of all of them was high. The clinical characteristics of the chest pain, age, past medical history of cardiovascular disease, gender, and abnormalities in the ECG were the predictors of CAD most commonly reported across the studies. The most recent papers, with highest methodological quality, and most practical for use in clinical settings, reported prediction or exclusion of CAD with area under the curve 0.90 in Primary Care, 0.91 in Emergency department, and 0.79 in Cardiology. These papers provide evidence of high level (1B) and the recommendation to use their results in the management of patients with chest pain is strong (A).

**Conclusions:**

The risk of CAD can be estimated on clinical grounds in patients with chest pain in different clinical settings with high accuracy. The estimation of probabilities of CAD presented in these studies could be used for a better management of patients with chest pain and also in the development of future predictive tools.

**Electronic supplementary material:**

The online version of this article (doi:10.1186/s12872-016-0196-4) contains supplementary material, which is available to authorized users.

## Background

Chest pain of recent onset is a common presenting symptom. In Primary Care up to 15 % of patients with chest pain have coronary artery disease (CAD), including angina pectoris and myocardial infarction, and this proportion increases to 22 % in Emergency departments and 28 % in Cardiology clinics [1–3]. However, the diagnosis of CAD among patients reporting chest pain remains difficult. Nonetheless, despite these acknowledged difficulties current guidelines state that clinical assessment alone may be sufficient to confirm or exclude the diagnosis of CAD in patients with stable chest pain [4–6]. Clinical history and physical examinations should be the first step of this clinical assessment and guide further diagnostic tests [4–6]. Many patients referred for further tests are now offered investigations which have high costs and some involve exposure to ionising radiation [4, 6]. Both unnecessary investigations and failure to diagnose CAD are important issues from a resource and clinical outcome perspective.

Predictive tools can help clinical decision making. Statistical models can accommodate a large number of factors and produce consistent results, improving the accuracy of clinical judgment, which is not as consistent, especially with less experienced clinicians [7]. Framingham, QRisk, and ASSIGN are validated risk predictive tools of cardiovascular disease (CVD), widely used in clinical practice, but they have not been validated in patients presenting with chest pain [8–10]. The recommendations included in the NICE guidelines for the clinical estimation of the risk of CAD in patients with stable chest pain, are based on a single study published in 1993 [4, 11]. However, it is acknowledged in the guidelines that this study may overestimate the risk of CAD in Primary Care, the setting where doctors usually have to base their decisions purely on the clinical assessment. More recent studies may also allow a more accurate estimation of the risk of CAD in different settings.

This systematic review aims to identify in the current literature predictive tools for clinical assessment of patients with chest pain, in primary and secondary care, and to assess their diagnostic performance. This evidence could inform both the management of patients with chest pain and the development of future diagnostic tools.

## Methods

The Meta-analysis Of Observational Studies in Epidemiology (MOOSE) criteria were used to undertake this review (Additional file 1) [12]. Searches were conducted in PUBMED, EMBASE, SCOPUS and the Web of Science, from database inception to the 31^st^ of July 2015.

We aimed to identify original studies in compliance with the following inclusion criteria:

1. Studies including patients with chest pain of recent onset
2. Studies using diagnosis of CAD as main outcome
3. Multivariate analysis to identify independent predictors of CAD
4. A predictive tool combining at least three predictors was derived
5. The predictive tool included variables from the history, physical examination, or electrocardiogram (ECG)
6. The predictive tool did not include variables from blood tests, cardiac stress testing, coronary angiography or coronary scans
7. A computer was not required by the clinician to estimate risk of CAD with the proposed tool

Studies were excluded if they were limited to specific patient characteristics (e.g. patients of a specific age group), or they included mixed exposure or outcome measures (e.g. chest pain or other symptoms in relation to CAD and pneumonia), unless separate results for chest pain and CAD were reported. Studies presenting computerized clinical decision support were not included in this review as our objective was to inform the decision taken by clinicians after clinical assessment, which is recommended to be the first approach to patients with chest pain, and the guide of all subsequent management. In some clinical settings, clinical history and physical examination may be the only assessment available [4–6].

The search strategy was defined with the help of a medical librarian and is presented in the Additional file 2. The titles and abstracts identified in the initial search were checked against inclusion criteria. A number of full text studies were selected and assessed for inclusion or exclusion. The bibliography of all relevant reviews identified in the initial search was also checked for further articles [13–25]. There were no restrictions on the basis of language, sample size or duration of follow-up. Relevant data was extracted from each study using a predefined template. Corresponding authors were contacted if any clarification was required. Two reviewers (LA and EG) independently selected the studies, assessed the quality and extracted all the data. Any discrepancies were resolved by consensus and by a third reviewer (VG).

The quality of the studies was assessed with the QUADAS tool [26]. Since the prevalence of CAD has changed in the past few decades the date of publication was acknowledged to be relevant in order to use the tool in clinical practice; more recent papers were considered more relevant [27, 28]. The predictive value of a tool is different for each clinical setting [29], therefore articles based in Primary Care, Emergency departments and Cardiology departments were assessed separately. The results from the studies were not summarised because it is difficult to produce sensible summaries using aggregate data meta-analysis when there are multiple different predictors used in each paper. The probabilities of CAD in patients with different clinical presentations, in each clinical setting, were estimated using the tool derived in the studies considered to be methodologically robust and epidemiologically up to date. For the Primary Care and Cardiology studies [30, 31], the predicted probabilities were calculated using the formula 1/(1 + e^-r^) where e is the base of natural logarithm and r is the linear predictor. For the Emergency department studies, one paper presented a tool to rule out CAD, which did not require any calculations [32], and another one presented a formula for calculation [33]. Details of the calculations are provided in Additional file 3. The authors of the studies used to calculate probabilities of CAD were contacted to make sure that a correct interpretation of their tool was being made. The evidence provided by the papers used to estimate probabilities of CAD was graded according to the levels of evidence proposed by the Oxford Centre for Evidence Based Medicine [34].

## Results

The initial search produced 19126 references from which 231 full text papers were assessed for inclusion. An additional nine full text studies were also assessed, from the 593 references of the reviews identified in the initial search. The first and second reviewer agreed in the inclusion or exclusion of 229 articles and the third reviewer assessed and decided over the inclusion of the 11 remaining articles. Finally, 12 papers were included in this review. All papers assessed in full text were in English except one, that was in Italian and was assessed by two reviewers (EG and VG) with knowledge of that language. Additional file 4 shows the number of references assessed at each stage.

Three studies had been conducted in Primary Care, (Table 1) [30, 35, 36] six in Emergency departments, (Table 2) [37–40] [32, 33] and four in Cardiology departments (Table 3) [11, 31, 35, 41]. One study assessed both patients from Cardiology and Primary Care [35]. The number of patients in the derivation samples ranged from 284 to 8176 with five studies including over a thousand patients [11, 30, 31, 35, 41]. All studies were conducted in the United States or Europe except one that was conducted in Brazil. The quality of the studies was high, with all of them scoring ≥9 in the 13 items assessment scale (Additional file 5) [26].

**Table 1:** Characteristics of the studies based in Primary Care

Author	Country	Patients for score derivation (n)	Outcome	Patients with outcome n (%)	Maximum time between assessment and outcome measure	Variables	Performance
Sox^a^ 1990 [35]	USA	1074	MI Angina Coronary insufficiency	424 (39.5)	1 year	Age Gender Exertional pain Patient stops activities when pain occurs PMH of MI Smoking Pain relived by NTG	For score > 4 S:0.99 Sp:0.18 PPV:0.45 NPV:0.98
Gencer 2010 [36]	Switzerland Germany	661	CAD	85 (12.9)	1 year	Age Gender CV Risk Known Pain location Pain duration Exertional pain Pain on palpation PMH of CVD	AUC: 0.95 (0.92-0.97)
							For scores < 5 (Lowest 5 % for patients with CAD):S:0.98 Sp:0.71 NPV:0.99
Bösner 2010 [30]	Germany Switzerland	1199	CAD	180 (15.0)	6 months	Age Gender Exertional pain PMH of CVD Patient assumes pain of cardiac origin Pain on palpation	AUC: 0.90 (0.87-0.93)
							For a cut-off value of 3 (risk of CAD > 35 %): S:0.86 (0.79-0.92) Sp:0.75 (0.72-0.78) PPV:0.35 (0.29-0.41) NPV:0.97 (0.96-0.98)

*MI* Myocardial Infarction, *PMH* Past Medical History, *NTG* Nitroglycerine, *AUC* Area Under the Curve, *S* Sensitivity, *Sp* Specificity, *PPV* Positive predictive value, *NPV* Negative predictive value

^a^This study included both patients from Cardiology and Primary Care

**Table 2:** Characteristics of the studies based in Emergency departments

Author	Country	Patients for score derivation (n)	Outcome	Patients with outcome n (%)	Maximum time from assessment to out come	Variables	Performance
Tierney 1985 [37]	USA	284	MI	35 (12.3)	3 months	Diaphoresis PMH of MI ST elevation Q wave	AUC: 0.85 For patients with 2 factors (risk of MI > 25 %): S:0.79 Sp:0.89 PPV:0.49 NPV:0.97
Grijeels 1995 [38]	Netherlands	815	MI Unstable Angina	400 (49.1)	At dis-charge	Gender Radiation of pain Nausea or sweating, PMH of CVD Abnormal ECG	AUC: 0.71
Goodacre 2002 [39]	UK	893	MI Cardiac death Arrhythmia Revascularisation	81 (9.1)	1 year	Radiation of pain Burning pain Nausea/vomiting Exertional pain Tender chest wall	Radiation of pain: PPV:0.14 (0.11-018) NPV:0.94 (0.91-0.95) Exertional pain: PPV: 0.17 (0.12-0.24) NPV: 0.92 (0.90-0.94)
Bassan 2004 [40]	Brazil	566	MI Unstable Angina	269 (47.5)	Outcome recorded during acute clinical management	Age Chest pain characteristics PMH of CAD Diabetes ST depression, T wave inversion	AUC: 0.90 (0.88-0.93) Using cut-off probability of CAD 10 % S: 0.99 Sp:0.41 PPV:0.60 NPV:0.98
Björk 2006 [33]	Sweden	634	MI Unstable Angina	130 (20.5)	Outcome recorded during acute clinical management	Age Hypertension Angina pectoris PMH of MI PMH of CABG Short symptoms duration Abnormal ECG	Derivation cohort AUC: 88.0 For S 0.95 Sp:0.5 PPV:0.33 NPV:0.98
Sánchez 2007 [32]	Spain	732	Not having CAD	533 (72.8)	1 month	Age Oppressive pain Pain location PMH of CAD Diabetes	AUC: 0.91 (0.89-0.93) S:0.17 Sp:1 PPV:1 NPV:0.31

*CABG* Coronary Artery Bypass Graft

**Table 3:** Characteristics of the studies based in Cardiology departments

Author	Country	Patients (n)	Out come	Patients with outcome	Maximum time between assessment and outcome measure	Variables	Performance
Sox^a^ 1990 [35]	USA	1074	MI Angina Coronay inssufficiency	424 (39.5)	1 year	Age Gender Exertional pain Patient stops activities when pain occurs PMH of MI Smoking Pain relived by NTG	For score > 4 S:0.99 Sp:0.18 PPV:0.45 NPV:0.98
Pryor 1993 [11]	USA	168	CAD	109 (64.9)	90 days	Age Gender Chest pain typicality PMH of MI Diabetes Smoking, Hyperlipidaemia ST-T wave changes Q Waves	AUC:0.87 (0.82-0.93)
Sekhri 2008 [41]	UK	8176	CAD	501 (6.1)	4 years	Age Gender Diabetes Chest pain typicality Bundle branch block Change in ST or T Q waves	For clinical assessment: AUC:0.73 (0.71-0.75) For clinical assessment +ECG: AUC:0.74 (0.72-0.76)
Genders 2012 [31]	USA Finland UK Hungary Austria Italy Russia Netherlands Belgium Germany Switzerland	5677	CAD	1634 (28.8)	Outcome recorded during acute clinical management	Age Gender Chest pain typicality Diabetes Hypertension Dislipaemia Smoking Body Mass Index	AUC:0.79 Net reclassification compared with model based only on age gender and typicality: 35 %

^a^This study included both patients from Cardiology and Primary Care

A number of outcomes were reported in different studies, which included CAD, myocardial infarction (MI), angina, cardiovascular death, and revascularisation interventions. Outcomes were defined using combinations of different methods including clinical presentation, ECG abnormalities, biochemical markers, need for revascularisation and diagnosis at discharge. The specific outcome used by each individual study is presented in Tables 1, 2 and 3. Most studies were conducted during the clinical management of patients and only one study clearly reported that the outcome measure was similar for all patients independently of their initial symptoms [35].

All studies derived the tools using predictors that were independently associated with the outcome in a multivariate regression model. One paper used cox regression, and reported associations of individual predictors as hazard ratios, and all the other papers used logistic regression and presented the associations as odd ratios. The clinical characteristics of the chest pain were associated with the outcome of 11 studies, five of them specifically reported exertional pain, and three reported typicality of chest pain, to be associated with the outcome. Age was associated with outcome in nine studies. Past medical history of cardiovascular disease was associated with outcome in nine studies. Gender in eight studies, abnormalities in the ECG in six, and symptoms associated with chest pain in two studies. However, the description of clinical symptoms, past medical history of cardiovascular disease, abnormalities of the ECG, and the symptoms associated with chest pain, were not recorded consistently across studies.

The three Primary Care studies reported reclassification of patients with different clinical presentation against specified thresholds. Two of them assessed the performance with measures of discrimination quantified with Area Under the receiver operating Curve (AUC) as well [30, 36]. These two studies also reported internal validation of their diagnostic tool with bootstrapping techniques, and validation in external populations in different countries, which showed in both cases, results consistent with the ones obtained in the main analysis [30, 36]. The study by Bösner et al. was the most recent, had the largest sample size, presented a clinical tool with the lowest number of variables, high predictive value, and the most consistent results in the derivation and validation [30]. The estimation of probabilities of CAD according to the diagnostic tool described in this study is presented in Table 4. This tool provides evidence graded as 1B and recommendation to use it in a Primary Care setting was graded A (maximum strength).

**Table 4:** Probabilities of Coronary Artery Disease (as percentages) in patients with chest pain in Primary Care

		Female <65 or Male <55				Female ≥65 or Male ≥55			
		Known CVD		No known CVD		Known CVD		No known CVD	
		Pain reproducible by palpation	Pain not reproducible by palpation	Pain reproducible by palpation	Pain not reproducible by palpation	Pain reproducible by palpation	Pain not reproducible by palpation	Pain reproducible by palpation	Pain not reproducible by palpation
Pain worse during exercise	Patient attributes pain to cardiac origin	21	63	5	24	48	86	15	53
	Patient attributes pain to cardiac origin	8	34	2	9	23	65	5	26
Pain not worse during exercise	Patient attributes pain to cardiac origin	5	25	1	6	16	55	3	19
	Patient attributes pain to cardiac origin	2	9	0	2	6	27	1	7

(Based on the tool by Bösner et al.) [30]. CVD: Clinical vascular disease

Note that probabilities are rounded to 0 decimal places, so a probability of 0 % does not imply impossibility

Five of the six studies conducted in Emergency departments had MI as an outcome, four of them had unstable angina as well, and one study used the probability of not having CAD as an outcome. The performance of the tool was reported with AUC in five studies [32, 33, 37, 38, 40], and measures of reclassification against specified thresholds were also provided in five studies [32, 33, 37, 39, 40]. Two studies measured the calibration of their tool with the Hosmer-Lemeshow test. [32, 37] One paper reported internal validation [33] and another two reported external validation in different populations [32, 37].

The study by Sánchez et al. [32] was the most recent one, and presented a tool to triage patients in the Emergency department and identify those who don’t need admission into a chest pain unit. In their observation all the patients in which the following five features were not present were confirmed not to have CAD:

- Age over 40
- Previously known CAD
- Diabetes
- Oppressive chest pain
- Retrosternal chest pain.

This model was validated in the same Emergency department four years later on 4231 patients and again 100 % (*n* = 231) of patients without any of the five features were confirmed not to have CAD [42]. This tool provides evidence graded as 1B and recommendation to use it in a clinical setting is of the maximum strength (grade A).

Another predictive tool, derived in an emergency department, considered to be methodologically robust and clinically relevant was developed by Björk et al. [33] It estimates the probability of CAD using nine variables providing an AUC 0.88. Björk et al. used a cross validation procedure and obtained results consistent with the ones observed in the derivation of the tool. It provides evidence graded as 1B and recommendation to use it in a clinical setting is of the maximum strength (grade A). An estimation of probabilities of CAD based on this tool is presented in Table 5. The full predictive model is presented in Additional file 3.

**Table 5:** Probabilities of Coronary Artery Disease (as percentages) in patients with chest pain in Emergency departments

		Age 40				Age 50				Age 60				Age 70			
		HTN+		HTN-		HTN+		HTN-		HTN+		HTN-		HTN+		HTN-	
Symp-toms dura- tion		Cd +	Cd-	Cd+	Cd-	Cd+	Cd-	Cd+	Cd–	Cd+	Cd-	Cd+	Cd-	Cd+	Cd-	Cd+	Cd-
0-6 h	AP+	26	16	13	8	33	22	18	11	42	28	24	15	50	36	31	20
	AP-	11	7	5	3	15	9	7	4	20	12	10	6	27	17	14	8
7-12 h	AP+	22	14	11	6	29	18	15	9	36	24	20	12	45	31	26	16
	AP-	9	5	4	2	13	7	6	3	17	10	8	5	23	14	11	7
>12 h	AP+	7	4	3	2	10	6	5	3	13	8	6	4	18	11	9	5
	AP-	3	1	1	1	4	2	2	1	5	3	2	1	7	4	3	2

(Based on the tool by Björk et al. [33])

Note: Estimations for patients without congestive heart failure, previous myocardial infarction, previous CABG or signs of acute coronary syndrome in the ECG

*HTN* Hypertension, *AP* pectoris within previous month, *Cd* Chest discomfort at arrival to hospital

One paper based in Cardiology department recruited participants from outpatient rapid access chest pain clinics [41], another one recruited participants referred for non invasive testing [11], one from angiography units [35], and the last one included both patients having CT coronary angiography and catheter based coronary angiography [31]. The performance of the tool was reported with AUC in three studies [11, 31, 41]. The study by Genders et al. [31] was the only one that reported measurements of calibration, together with the measures of discrimination and reclassification, of its predictive tool. It was also the most recent and was conducted in 18 hospitals in 11 different countries. It included patients with symptoms suggestive of stable angina undergoing angiography. It was also the only study in which the predictive tool was validated (using cross validation), and results were consistent with the ones obtained in the derivation. The estimation of probabilities of CAD according to the prediction rule described in this study is presented in Table 6. This tool provides evidence graded as 1A and recommendation to use it in a clinical setting of the maximum strength.

**Table 6:** Probabilities of Coronary Artery Disease (as percentages) in patients with symptoms suggestive of stable angina undergoing angiography

Female				Age 40			Age 50			Age 60			Age 70			Age 80		
				T	A	Ns	T	A	Ns	T	A	Ns	T	A	Ns	T	A	Ns
DM+	HTN+	Dislip+	Smk+	20	8	5	31	15	8	46	24	15	61	37	24	74	53	37
			Smk-	13	6	3	22	10	5	35	17	10	50	27	17	65	41	27
		Dislip-	Smk+	14	6	3	23	10	6	36	17	10	51	28	17	66	42	28
			Smk-	9	4	2	16	7	4	26	12	7	39	20	12	55	31	20
	HTN-	Dislip+	Smk+	15	6	3	24	11	6	37	19	11	53	30	18	67	44	30
			Smk-	10	4	2	17	7	4	27	13	7	41	21	12	57	33	21
		Dislip-	Smk+	10	4	2	17	7	4	28	13	7	42	22	13	58	34	22
			Smk-	7	3	1	12	5	3	20	9	5	32	15	9	46	25	15
DM-	HTN+	Dislip+	Smk+	14	4	2	23	7	4	35	12	7	50	21	12	65	33	20
			Smk-	9	2	1	16	5	2	26	8	4	39	14	8	54	23	14
		Dislip-	Smk+	9	3	1	16	5	3	26	8	5	40	15	8	55	24	14
			Smk-	6	2	1	11	3	2	18	5	3	30	10	5	44	17	10
	HTN-	Dislip+	Smk+	10	3	2	17	5	3	28	9	5	42	16	9	57	26	15
			Smk-	7	2	1	12	3	2	20	6	3	31	10	6	46	18	10
		Dislip-	Smk+	7	2	1	12	3	2	20	6	3	32	11	6	47	18	11
			Smk-	4	1	1	8	2	1	14	4	2	23	7	4	36	12	7
Male																		
DM+	HTN+	Dislip+	Smk+	48	26	16	63	40	26	76	55	39	86	69	55	92	81	69
			Smk-	37	18	11	52	29	18	67	43	29	79	59	43	87	73	58
		Dislip-	Smk+	38	19	11	53	30	19	68	44	30	80	60	44	88	73	59
			Smk-	28	13	7	41	21	13	57	33	21	71	48	33	82	63	48
	HTN-	Dislip+	Smk+	40	20	12	55	32	20	69	46	32	81	62	46	89	75	61
			Smk-	29	14	8	44	23	14	59	35	22	73	50	35	83	65	50
		Dislip-	Smk+	30	14	8	44	23	14	60	36	23	73	51	36	84	66	51
			Smk-	21	9	5	34	16	9	48	26	16	64	40	26	76	55	40
DM-	HTN+	Dislip+	Smk+	38	13	8	53	22	13	68	35	22	79	50	34	88	65	49
			Smk-	28	9	5	41	15	9	57	25	15	71	38	25	82	54	38
		Dislip-	Smk+	28	9	5	42	16	9	58	26	16	72	39	26	82	55	39
			Smk-	20	6	3	32	11	6	46	18	10	62	29	18	75	43	29
	HTN-	Dislip+	Smk+	30	10	5	44	17	10	60	27	17	73	41	27	84	57	41
			Smk-	21	6	4	33	11	6	48	19	11	63	31	19	76	45	30
		Dislip-	Smk+	22	7	4	34	12	7	49	20	12	64	32	20	77	46	31
			Smk-	15	4	2	25	8	4	38	14	8	53	23	13	68	35	22

(Based on the tool by Genders et al.) [31]

*DM* Diabetes Mellitus, *HTN* Hypertension, *Dislip* Dislipaemia, *Smk* Smoking (positive if the patient is a current smoker or an ex-smoker), *T* Typical chest pain, *A* Atypical chest pain, *Ns* Non-specific chest pain

## Discussion

CAD can be confirmed or ruled out on clinical grounds in patients with chest pain in Primary Care, Emergency departments and Cardiology clinics with levels of discrimination up to 91 %, based on AUC. The clinical characteristics of the chest pain, age, past medical history of cardiovascular disease, gender, and abnormalities in the ECG were the predictors of CAD most commonly reported across the studies.

The comprehensive search and critical assessment of articles, conducted by three reviewers, following standard guidelines for reviews of observational research, represents a strength of this study [12]. It provides strong evidence of the performance, including discrimination calibrations and reclassification measures, of the diagnostic tools to predict CAD in different clinical settings. The diversity of the methods used across studies may have an effect on the external validity of each individual one.

Previous systematic reviews on the value of the clinical assessment of patients with suspected CAD have focused on studies based in Emergency departments [17, 24, 43], have reported the prognostic value of individual signs and symptoms [13, 14, 23], or include predictors not available on clinical history or examination, such as biomarkers [17, 18]. However, clinicians working in primary and secondary care base their decisions on combinations of signs and symptoms and in many cases are not supported by laboratory or radiology tests. The results of this study complement a review that assessed scores for CAD that included laboratory tests. They presented TIMI and GRACE scores, as the most extensively investigated, with GRACE showing better performance, and reported that there are other potentially useful risk scores that had not been validated [18].

Future studies may derive diagnostic tool, or validate [44] the ones presented in this review, in larger datasets and use more uniform measures for predictors and outcomes. Ideally the outcome to be used in future studies should include all clinically relevant cases of CAD rather than specific ones such as only angina or only MI. The performance of future predictive tools should also include measures of discrimination, calibration, which was only reported in three papers included in this review [31, 32, 37], and reclassification [45]. Measures of impact, that is whether the predictive tool is used by physicians, changes therapeutic decisions, improves the clinical process and patient outcome, or reduces costs, should also be reported [44]. As computers become more available, and use of software more accepted in clinical settings [46, 47], further research may also look at computer aided clinical decision support tools for patients with chest pain of recent onset.

The guidelines from the European Society of Cardiology acknowledges that non-invasive, imaging-based diagnostic methods for CAD have typical sensitivities and specificities of approximately 85 %, therefore 15 % of all diagnostic results will be false. For this reason they recommend no testing if the probabilities of CAD estimated on clinical grounds are <15 % or >85 %, assuming that these patients are healthy or have stable CAD respectively [6]. The NICE, and the American Heart Association, guidelines use thresholds for further tests between 10 % and 90 % [4, 5]. Authors of future predictive tools may therefore consider reporting measures of reclassification against these defined, non arbitrary, thresholds for clinical intervention [45], which were only used in one study included this review [40].

## Conclusions

The predictors of CAD observed in our review may be considered by clinicians, and also by researchers in the development of further diagnostic tools. The estimated probability of CAD presented in this review can be used in the assessment of patients with chest pain in primary care, emergency departments and cardiology clinics. However, despite the good performance of some of the predictive tools, none can guarantee a completely accurate diagnosis of CAD, therefore clinical judgement remains an essential element for the clinical decision.

## Additional files

Additional file 1:
**MOOSE statement.** (DOCX 16 kb) Additional file 2:
**Search strategy.** (DOCX 13 kb) Additional file 3:
**Prediction Models.** (DOCX 22 kb) Additional file 4:
**Number of references assessed for inclusion.** (DOCX 33 kb) Additional file 5:
**Methodological quality of studies included in the review** ^**26**^ **.** (DOCX 16 kb)

## Abbreviations

CAD: Coronary artery disease
AUC: Area Under the receiver operating Curve
