# Supplementary material for: Clinical assessment of patients with chest pain; a systematic review of predictive tools
Source: BMC Cardiovasc Disord. 2016 Jan 20;16:18. doi: 10.1186/s12872-016-0196-4 (PMC4721048; doi:10.1186/s12872-016-0196-4)
Supplement: Additional file 1: — MOOSE statement. (DOCX 16 kb) [file 12872_2016_196_MOESM1_ESM.docx]

**Supplement 1. MOOSE statement**

| Reporting Criteria | Reported | Page |
| --- | --- | --- |
| Reporting of Background | Yes | 6 |
| Problem definition | Yes | 6 |
| Hypothesis statement | yes | 6 |
| Description of Study Outcome(s) | Yes | 6 |
| Type of exposure or intervention used | Yes | 6 |
| Type of study design used | Yes | 6 |
| Study population | Yes | 6 |
| Reporting of Search Strategy | Yes | Supp. 2 |
| Qualifications of searchers | Yes | 1, 7 |
| Search strategy, including time period included in the synthesis and keywords | Yes | 7 |
| Effort to include all available studies, including contact with authors | Yes | 8 |
| Databases and registries searched | Yes | 7 |
| Search software used, | - | - |
| Use of hand searching (eg, reference lists of obtained articles) | Yes |  |
| List of citations located and those excluded, including justification | Yes | Supp. 4 |
| Method for addressing articles published in languages other than English | Yes | 9 |
| Method of handling abstracts and unpublished studies | NA | - |
| Description of any contact with authors | Yes | 8 |
| Reporting of Methods | Yes | 7-9 & Supp. 3 |
| Description of relevance or appropriateness of studies assembled for assessing the hypothesis to be tested | Yes | 8 |
| Rationale for the selection and coding of data | Yes | 8 |
| Documentation of how data were classified and coded | Yes | 8 |
| Assessment of confounding | Yes | 7 |
| Assessment of study quality, including blinding of quality assessors;  stratification or regression on possible predictors of study results | Yes | 8  Supp 5. Ref 25 & 26 |
| Assessment of heterogeneity | NA | - |
| Description of statistical methods in sufficient detail to be replicated | Yes | 8 & Supp. 3 |
| Provision of appropriate tables and graphics | Yes | 10,11,12,14,16,18 |
| Reporting of Results | Yes | 9-16 |
| Table giving descriptive information for each study included | Yes | 10,11,12 |
| Results of sensitivity testing (eg, subgroup analysis) | Yes | 14, 16, 18 |
| Indication of statistical uncertainty of findings | Yes | 10, 11, 12 |
| Reporting of Discussion | Yes | 19, 20, 21 |
| Quantitative assessment of bias (eg, publication bias) | NA | - |
| Justification for exclusion (eg, exclusion of non–English-language citations) | Yes | Supp. 4 |
| Assessment of quality of included studies | Yes | Supp. 5 |
| Reporting of Conclusions | Yes | 19, 20, 21 |
| Consideration of alternative explanations for observed results | NA | - |
| Generalization of the conclusions (ie, appropriate for the data presented and  within the domain of the literature review) | Yes | 20 |
| Guidelines for future research | Yes | 20, 21 |
| Disclosure of funding source | Yes | 21 |
