# Supplementary material for: Clinical assessment of patients with chest pain; a systematic review of predictive tools
Source: BMC Cardiovasc Disord. 2016 Jan 20;16:18. doi: 10.1186/s12872-016-0196-4 (PMC4721048; doi:10.1186/s12872-016-0196-4)
Supplement: Additional file 2: — Search strategy. (DOCX 13 kb) [file 12872_2016_196_MOESM2_ESM.docx]

**Supplement 2. Search strategy**

1- Chest Pain

2- Typical

3- Atypical

4- Location

5- Severity

6- Duration

7- Exercise

8- Physical Exertion

9- Rest

10- Emotions

11- Nitrates

12- Epidemiologic Factors

13- Myocardial Ischemia

14- Acute myocardial infarction

15- Coronary atherosclerosis

16- Coronary arteriosclerosis

17- Coronary obstruction

18- Coronary Artery Atherosclerosis

19- Coronary artery arteriosclerosis

20- Coronary Artery Obstruction

21- Ischaemic cardiomyopathy

22- Ischaemic heart disease

23- Prinzmetal's angina

24- Coronary artery spasm

25- Coronary spasm

26- Predictive Value of Tests

27- Risk Assessment

28- Medical History Taking

29- Diagnosis

30- 2 OR 3 OR 4 OR 5 OR 6 OR 7 OR 8 OR 9 OR 10 OR 11 OR 12

31- 1 AND 30

32- 13 OR 14 OR 15 OR 16 OR 17 OR 18 OR 19 OR 20 OR 21 OR 22 OR 23 OR 24 OR 25

33- 26 OR 27 OR 28 OR 29

34- 31 AND 32 AND 33
