# Supplementary material for: Clinical assessment of patients with chest pain; a systematic review of predictive tools
Source: BMC Cardiovasc Disord. 2016 Jan 20;16:18. doi: 10.1186/s12872-016-0196-4 (PMC4721048; doi:10.1186/s12872-016-0196-4)
Supplement: Additional file 3: — Prediction Models. (DOCX 22 kb) [file 12872_2016_196_MOESM3_ESM.docx]

**Supplement 3: Prediction Models**

*Table 4 based on Bösner* *et al.*^30^

The probability of coronary artery disease was calculated as

1/(1+e^-r^)

where e = base of natural logarithm;

r = β_0_ + β_1_ x_1_ + β_2_ x_2_ +…+ β_k_ x_k_

where β_0_ is the intercept term (in this case -5.787)

x_1_, x_2_,…, x_k_ are the characteristics

β_1_, β_2_,…, β_k_ are the corresponding logistic regression coefficients

The characteristics are listed below with their coefficients:

Characteristics Coefficient

Age/Sex (female ≥ 65, male ≥ 55) 1.286

Known clinical vascular disease* 1.666

Pain worse during exercise 1.612

Pain not reproducible by palpation 1.860

Patient assumes pain is of cardiac origin 1.161

Note that the variables took value 1 if the statement concerning the variable was true, and 0 otherwise. *Coronary artery disease, occlusive disease or cerebrovascular disease.

*Table 5 based on Björk et al.^33^*

The probability of ACS was calculated as

A/(1+A)

where  A = estimated odds of ACS = α_0_ * α_1_^(age-40)^* α_2_ *…* α_k_

where α_0_ is the baseline odds (in this case 0.0066)

            α_1_ is the odds ratio corresponding to the age variable (in this case 1.036)

            α_2_,…, α_k_ are the odds ratios for the other risk factors of the patient

The risk factors are listed below with their odds ratios:

Risk factors Odds ratio

Age (no. of years above 40) 1.036

Hypertension 2.3

Angina pectoris ≤ 1 month 2.8

Congestive heart failure 0.55

Previous myocardial infarction

Yes, ≤ 6 months 3.4

Yes, > 6 months 1.9

No 1.0

Previous CABG 0.28

Chest discomfort at presentation 1.8

Symptom duration

0-6 h 4.6

7-12 h 3.7

> 12 h 1.0

ECG expert assessment

ACS and TMI 97

ACS but not TMI 11

Probably ACS 5.8

No signs of ACS 1.0

ACS: acute coronary syndrome. TMI: transmural ischaemia

For example, the probability of CAD for a a 72 year old patient reporting chest pain lasting eight hours, with medical history of coronary artery bypass graft, previous MI more than 6 months ago, angina pectoris in the previous month and an ECG showing ACS and TMI would be calculated as follows:

0.0066 x 1.036^(72-40)^ x 3.7 x 0.28 x 1.9 x 2.8 x 97 = 10.9420

Probability= 10.9420/(1+10.9420) = 0.92 (2 d.p.)

*Table 6 based on Genders et al.* ^31^

The probability of coronary artery disease was calculated as

1/(1+e^-r^)

where e = base of natural logarithm;

r = β_0_ + β_1_ x_1_ + β_2_ x_2_ +…+ β_k_ x_k_

where β_0_ is the intercept term (in this case -7.539)

x_1_, x_2_,…, x_k_ are the characteristics

β_1_, β_2_,…, β_k_ are the corresponding logistic regression coefficients

The characteristics are listed below with their coefficients:

Characteristics Coefficient

Age 0.062

Sex (1 = male, 0 = female) 1.332

Atypical chest pain (1 if present) 0.633

Typical chest pain (1 if present) 1.998

Diabetes (1 if present) 0.828

Hypertension (1 if present) 0.338

Dyslipidaemia (1 if present) 0.422

Smoking (1 if smoking, 0 if non-smoking) 0.461

Diabetes * Typical chest pain (interaction) -0.402

Note that the clinical model also included a variable for ‘setting’ to account for differences in patient selection across datasets, but when applying the model for new patients this setting variable is set to zero (low prevalence setting). The model also included a random effect for hospital, but predicted probabilities were calculated using the fixed part of the model only.
