# Supplementary material for: Clinical assessment of patients with chest pain; a systematic review of predictive tools
Source: BMC Cardiovasc Disord. 2016 Jan 20;16:18. doi: 10.1186/s12872-016-0196-4 (PMC4721048; doi:10.1186/s12872-016-0196-4)
Supplement: Additional file 4: — Number of references assessed for inclusion. (DOCX 33 kb) [file 12872_2016_196_MOESM4_ESM.docx]

**Supplement 4: Number of references assessed for inclusion**

Results from Initial electronic search:

PubMed: 10123

EMBASE: 5507

SCOPUS: 1752

Web of Science: 1744

Studies from references lists of reviews identified in the electronic search: 593

Full text studies assessed for inclusion: 9

Full text studies assessed for inclusion: 231

Studies excluded: 228

- Aiming to identify other diseases

- Studies including only men or women

- Including variables from laboratory,

exercise ECG, or angiography

- Presenting analysis of observations

reported in another paper

- No multivariate analysis

- Studies testing tools derived in a different

publication

- Reporting predictive value of individual

variables

- Deriving a predictive tool that needs a

computer to be used

- Participants not reporting symptoms,

addressed by researchers with a

questionnaire.

Studies included in the review: 12
