# Supplementary material for: Clinical assessment of patients with chest pain; a systematic review of predictive tools
Source: BMC Cardiovasc Disord. 2016 Jan 20;16:18. doi: 10.1186/s12872-016-0196-4 (PMC4721048; doi:10.1186/s12872-016-0196-4)
Supplement: Additional file 5: — Methodological quality of studies included in the review 26 . (DOCX 16 kb) [file 12872_2016_196_MOESM5_ESM.docx]

**Supplement 5: Methodological quality of studies included in the review^26^**

|  | Tierney  1985 | Sox  1990 | Pryor 1993 | Grijeels  1995 | Good acre  2002 | Bassan  2004 | Björk  2006 | Sán  chez 2007 | Sekhri 2008 | Bösner 2010 | Gencer 2010 | Gen  ders 2012 |
| --- | --- | --- | --- | --- | --- | --- | --- | --- | --- | --- | --- | --- |
| Was the spectrum of patients representative of the patients who will receive the test in practice? | Y | Y | Y | Y | Y | Y | Y | Y | Y | Y | Y | Y |
| Were selection criteria clearly described? | Y | Y | Y | Y | Y | Y | Y | Y | Y | Y | Y | Y |
| Is the reference standard likely to correctly classify the target condition? | Y | Y | Y | Y | Y | Y | Y | Y | Y | Y | Y | Y |
| Is the time period between reference standard and index test short enough to be reasonably sure that the target condition did not change between the two tests? | Y | Y | Y | Y | Y | Y | Y | Y | Y | Y | Y | Y |
| Did the whole sample or a random selection of the sample, receive verification using a reference standard of diagnosis? | Y | Y | Y | Y | Y | Y | Y | Y | Y | Y | Y | Y |
| Did patients receive the same reference standard regardless of the index test result? | N | Y | N | N | N | N | N | N | N | N | N | N |
| Was the execution of the index test described in sufficient detail to permit replication of the test? | Y | Y | Y | Y | Y | Y | Y | Y | Y | Y | Y | Y |
| Was the execution of the reference standard described in sufficient detail to permit its replication? | Y | Y | Y | Y | Y | Y | N | Y | Y | Y | Y | Y |
| Were the index test results interpreted without knowledge of the results of the reference standard? | Y | Y | Y | Y | Y | Y | Y | Y | Y | Y | Y | Y |
| Were the reference standard results interpreted without knowledge of the results of the index test? | N | N | N | N | N | N | N | N | N | N | N | N |
| Were the same clinical data available when test results were interpreted as would be available when the test is used in practice? | Y | Y | Y | Y | Y | Y | Y | Y | Y | Y | Y | Y |
| Were uninterpretable/ intermediate test results reported? | Y | Y | Y | Y | Y | Y | Y | Y | Y | Y | Y | Y |
| Were withdrawals from the study explained? | N | N | N | Y | Y | N | N | Y | Y | Y | Y | Y |

Y: Yes

N: No
